# The Role of GST Gene Polymorphic Variants in Antipsychotic-Induced Metabolic Disorders in Schizophrenia: A Pilot Study

**DOI:** 10.3390/ph18070941

**Published:** 2025-06-21

**Authors:** Irina A. Mednova, Ekaterina V. Mikhalitskaya, Natalia M. Vyalova, Diana Z. Paderina, Dmitry A. Petkun, Vladimir V. Tiguntsev, Elena G. Kornetova, Nikolay A. Bokhan, Svetlana A. Ivanova

**Affiliations:** 1 Mental Health Research Institute, Tomsk National Research Medical Center, Russian Academy of Sciences, Tomsk 634014, Russia; irinka145@yandex.ru (I.A.M.); uzen63@mail.ru (E.V.M.); natarakitina@yandex.ru (N.M.V.); osmanovadiana@mail.ru (D.Z.P.); substantia_p@mail.ru (D.A.P.); cristall2009@live.ru (V.V.T.); ekornetova@outlook.com (E.G.K.); bna909@gmail.com (N.A.B.); 2 Department of Psychiatry, Narcology and Psychotherapy, Siberian State Medical University, Tomsk 634050, Russia

**Keywords:** *GSTP1*, metabolic syndrome, BMI, schizophrenia, polymorphic variants, pharmacogenetics, antipsychotics

## Abstract

The life expectancy of patients with psychotic disorders is significantly shorter than that of the general population; antipsychotic-induced metabolic disorders play a significant role in reducing life expectancy. Both metabolic syndrome (MetS) and schizophrenia are multifactorial conditions. One area where the two conditions overlap is oxidative stress, which is present in both diseases. The glutathione-S-transferase (GST) system is a major line of defense against exogenous toxicants and oxidative damage to cells. The aim of our study was to perform an association analysis of gene polymorphisms with metabolic disorders in patients with schizophrenia treated with antipsychotic therapy. **Methods**: A total of 639 white patients with schizophrenia (ICD-10) from Siberia (Russia) were included in the study. Genotyping was carried out using real-time polymerase chain reaction for two single-nucleotide polymorphisms (SNPs) in the *GSTP1* (rs614080 and rs1695) and one SNP in the *GSTO1* (rs49252). **Results**: We found that rs1695*GG genotype of *GSTP1* is a risk factor for the development of overweight (OR 2.36; 95% CI: 1.3–4.29; *p* = 0.0054). In the subgroup of patients receiving first-generation antipsychotics as basic therapy, the risk of overweight was associated with carriage of the rs1695*GG (OR 5.43; 95% CI: 2.24–13.16; *p* < 0.001) genotype of *GSTP1* in a recessive model of inheritance. In contrast, an association of rs1695*G GSTP1 with obesity (OR: 0.42; 95% CI: 0.20–0.87; *p* = 0.018) was shown in the dominant model of inheritance in patients receiving second-generation antipsychotics. **Conclusions**: The pilot results obtained confirm the hypothesis of a violation of the antioxidant status, in particular the involvement of *GSTP1,* in the development of antipsychotic-induced metabolic disorders in schizophrenia. Further studies with larger samples and different ethnic groups are needed to confirm the obtained results.

## 1. Introduction

Schizophrenia is a complex mental disorder that affects not only the cognitive and emotional aspects of patients but also their physical health. In recent years, there have been increasing findings regarding a high prevalence of metabolic disorders, such as obesity, type 2 diabetes, and dyslipidemia, in people with schizophrenia [1,2,3,4]. According to various data, the frequency of metabolic syndrome (MetS) varies widely, and, in the population of patients with schizophrenia, it is 10.1–42.7% versus 6–33.9% in the general population [5,6,7,8,9,10,11,12]. The wide range of data on the frequency of MetS occurrence may be due to the presence of various diagnostic criteria and differences in incidence among ethnic and age groups. A recent meta-analysis including the results of 12 studies involving 1953 participants found that the prevalence of MetS among patients with schizophrenia was 41.3% [13]. Obesity prevalence in schizophrenia patients is also 1.42 to 3.5 times higher than in the general population [14,15]. Cameron et al. (2017), in a large-scale study including 4658 schizophrenia cases and 19,686 healthy individuals, report that 31.6–36% of schizophrenia patients are obese by body mass index (BMI) criteria, compared with 19.5–25.4% in matched controls [15]. The increased prevalence of metabolic dysfunction in people with schizophrenia can be attributed, but is not limited to, to a number of factors, including poor diet, cigarette smoking, lack of exercise, stress, dysfunction of the hypothalamic–pituitary–adrenal axis, and use of antipsychotic medication [16]. Antipsychotic-induced metabolic disorders can significantly reduce the quality of life of patients [17], be a reason for noncompliance [18,19], and increase the risk of developing concomitant cardiometabolic diseases [20]. Risk factors for MetS in patients with schizophrenia include antipsychotic therapy and illness duration, and, in some studies, gender; however, metabolic abnormalities have also been reported in drug-naïve patients with first-episode psychosis [5,6,7,8,11]. Therefore, genes may play a role in metabolic dysregulation in patients with schizophrenia. According to case-control association studies, a number of genetic variants are potentially responsible for the high comorbidity between metabolic abnormalities and schizophrenia [21]. Majority studies focused on target genes involved in antipsychotic drug action and weight regulation, but genes responsible for other biological pathways are being identified [22,23,24].

A growing body of research has recently focused on the role of oxidative stress in the pathogenesis of obesity and metabolic syndrome in the general population. Excess adipose tissue, especially visceral, promotes the formation of proinflammatory cytokines, which leads to the activation of macrophages and increased production of free radicals [25]. This creates a vicious cycle; inflammation causes oxidative stress, and oxidative stress worsens inflammation, which ultimately contributes to the development of MetS and its associated complications [25,26]. Oxidative stress causes mitochondrial dysfunction, protein damage, lipid peroxidation, and impairing antioxidant function [26]. This also leads to dysregulation of adipocytokine production, which contributes to the development of obesity-associated vasculopathy and cardiovascular risk due to endothelial dysfunction [27]. Metabolic disorders are accompanied by a decrease in glutathione levels and changes in the activity of glutathione metabolism enzymes, the first line of defense against oxidative stress [28,29,30].

Conversely, a large body of research suggests that oxidative stress may be one of the mechanisms underlying the pathophysiology of schizophrenia. While it is not considered the primary cause of the disease, it can be incorporated into most current schizophrenia hypotheses and may play an important role in unifying these in the future [31]. It is suggested that oxidative stress may contribute to the progression of the disease and poor outcomes [32]. The association between schizophrenia and oxidative stress is hypothesized to be influenced by inflammatory and autoimmune regulation processes, neurotransmitters, such as dopamine, glutamate, and nitric oxide, mitochondrial and metal metabolism, and genetic and epigenetic factors [33,34]. A systematic review revealed that people with schizophrenia have a deficiency of glutathione and disturbances in the glutathione redox cycle [35]. Thus, oxidative stress may be the point of contact between MetS and schizophrenia.

Genetic variations in antioxidant defense genes and reactive oxygen species producing enzymes may influence the risk of obesity and related metabolic complications [36]. The glutathione S-transferase (GST) supergene family plays a pivotal role in antioxidant defense mechanisms, detoxifying electrophilic xenobiotics, and inactivating a variety of endogenous products with reduced glutathione [37,38]. The human cytosolic GST superfamily currently encompasses a minimum of 16 genes, which are divided into eight distinct classes, designated as follows: Alpha, Mu, Pi, Theta, Zeta, Sigma, and Omega [39]. A substantial body of research has characterized human GSTs, which have been found to exhibit polymorphism at varying frequencies based on ethnicity. The *GSTP1* gene has several polymorphic variants, of which the most functionally significant is rs1695, which results in the substitution of isoleucine for valine at codon 105 (Ile105Val). Functional genomic studies in COS-1 cells have revealed that rs1695 is one of several polymorphisms that can significantly alter *GSTP1* enzyme activity, protein levels, and substrate affinity; this may affect GSTP1′s role in drug metabolism and, potentially, disease pathogenesis and/or drug response [40]. The rs4925 polymorphism in *GSTO1* results in a substitution of alanine for aspartate at codon 140 (Ala140Asp). This polymorphism influences the kinetics of deglutathionylation, thioltransferase, and glutathionylation reactions [41,42].

The present study investigated the possible associations between three polymorphisms in *GSTP1* and *GSTO1* genes and the risk of overweight and MetS in patients with schizophrenia.

## 2. Results

### 2.1. Association of Studied SNPs with MetS, Anthropometric and Laboratory Parameters in the Overall Group of Patients

We studied two single nucleotide polymorphisms (SNPs) in the *GSTP1* (rs614080 and rs1695) and one SNP in the *GSTO1* (rs49252). All SNPs studied were in Hardy–Weinberg equilibrium. Characteristics of studied SNPs are presented in Table S1. We found no associations between the studied SNPs and MetS (Table S2). We found statistically significant differences in the frequencies of rs1695 genotypes in the *GSTP1* between normal and overweight schizophrenia patients (χ^2^ = 6.749, *p* = 0.034; Table 1).

Two models, codominant and recessive, were statistically significant. However, the information criteria (Akaike and Bayesian) are the lowest for the recessive model, which defines it as the best model. Consequently, the GG genotype carriage was identified as a risk factor for overweight in patients with schizophrenia (OR = 2.36; 95% CI: 1.30–4.29; *p* = 0.0054) (Table 2). There was no interaction between the SNP and the covariates (gender and smoking) (Table S3).

We found that the carrying of *GSTP1* rs614080*GG is associated with higher total cholesterol (*p* = 0.046) and low-density lipoprotein (*p* = 0.028) levels. No other differences in lipid and glucose levels were found in carriers of different genotypes of the genes studied (Table 3).

The carrying of *GSTP1* rs1695*GG is associated with higher abdominal fat fold (*p* = 0.023; Table 4).

### 2.2. Association of Studied SNPs with MetS, Anthropometric and Laboratory Parameters in the Group of Patients Receiving First-Generation Antipsychotics

First-generation antipsychotics (FGAs) and second-generation antipsychotics (SGAs) have different effects on the development of MS; metabolic disorders more often develop after taking atypical antipsychotics [8,38,43]. Although the data are inconclusive, FGAs have been shown to predominantly have prooxidant properties. In contrast, SGAs do not significantly affect redox processes or exhibit antioxidant activity [34,44]. Because the sample consisted of patients with chronic schizophrenia who had received long-term antipsychotic therapy, we analyzed the associations of the selected SNPs with metabolic parameters depending on whether the patients received FGAs or SGAs as part of their basic therapy.

In the group of patients who received FGAs, we also found no associations between the studied SNPs and MetS (Table S4). We found statistically significant differences in the frequencies of rs1695 genotypes (χ^2^ = 13.793; *p* = 0.001) and alleles (χ^2^ = 4.976; *p* = 0.026) in the *GSTP1* between normal and overweight schizophrenia patients (Table 5).

The risk of overweight was associated with carriage of the rs1695*GG (OR 5.43; 95% CI: 2.24–13.16; *p* < 0.001) genotype in a recessive model of inheritance (Table 6). There was no interaction between the SNP and the covariates (gender and smoking) (Table S3).

We did not find significant associations of the studied SNPs with blood lipids and glucose (Table S5). The carrying of *GSTP1* rs1695*GG is associated with higher abdominal fat fold (*p* = 0.037) and visceral fat level (*p* = 0.047; Table 7).

### 2.3. Association of Studied SNPs with MetS, Anthropometric and Laboratory Parameters in the Group of Patients Receiving Second-Generation Antipsychotics

In the group of patients who received SGAs, we also found no associations between the studied SNPs and MetS (Table S6). We found statistically significant differences in the frequencies of rs1695 alleles (χ^2^ = 5.87; *p* = 0.015) in the *GSTP1* between normal and obese schizophrenia patients (Table 8).

The dominant model of inheritance showed an association between rs1695 in *GSTP1* and obesity (OR: 0.42, 95% CI: 0.20–0.87; *p* = 0.018; Table 9). There was no interaction between the SNP and the covariates (gender and smoking) (Table S3).

The carrying of *GSTP1* rs614080*GG is associated with higher total cholesterol (*p* = 0.015) and low-density lipoprotein (*p* = 0.036) levels (Table 10). We did not find significant associations of the studied SNPs with anthropometric parameters (Table S7).

## 3. Discussion

**Figure 1:**
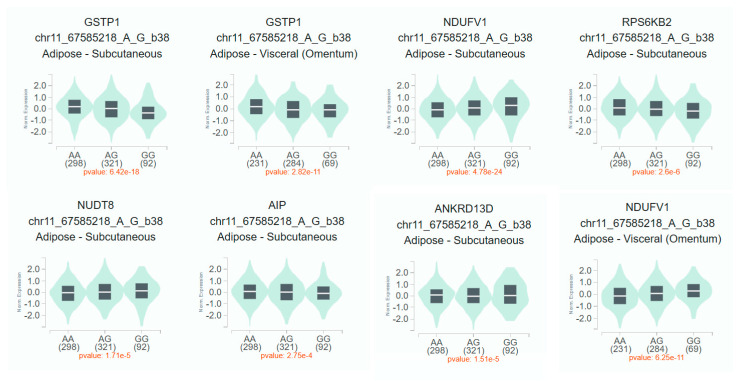
Tissue-specific eQTL analysis for *GSTP1* rs1695 polymorphism. Notes. The square boundaries are the 1st and 3rd quartiles, the line inside the square is the median.

We found associations of polymorphic variants rs1695 and rs614080 of the *GSTP1* gene with metabolic buildups in patients with schizophrenia. No associations were found for the rs4925 polymorphic variant *GSTO1*. *GSTP1* is widely expressed in various tissues of the human body, including adipose tissue [45]. According to the GeneOntology database, in addition to the implementation of biological processes associated with glutathione and xenobiotic metabolism, GSTP1 is involved in long-chain fatty acid biosynthesis processes [46]. According to the results of the association analysis, only the *GSTP1* rs1695 polymorphic variant showed statistically significant results. We used the GTEx Portal database (https://gtexportal.org; accessed on 10 May 2025) [47] to evaluate the tissue-specific effects of the G minor allele of the studied SNP. The results of tissue-specific eQTL analysis for *GSTP1* rs1695 polymorphism are presented in Figure 1.

The rs1695-G allele was associated with decreased *GSTP1* gene expression in subcutaneous (*p* = 6.42 × e^−18^) and visceral (*p* = 2.82 × e^−11^) adipose tissue. Meanwhile, this allele was associated with increased mRNA levels of *NDUFV1* genes in subcutaneous (*p* = 4.78 × e^−24^) and visceral (*p* = 6.25 × e^−11^) adipose tissue and *NUDT8* in subcutaneous adipose tissue (*p* = 1.71 × e^−5^). Carriage of the rs1695-G allele was also associated with decreased expression of *RPS6KB2* (*p* = 2.6 × e^−6^), *AIP* (*p* = 2.75 × e^−4^), and *ANKRD13D* (*p* = 1.5 × e^−5^) genes in subcutaneous adipose tissue. Thus, there is sufficient convincing evidence regarding the probable involvement of the rs1695 *GSTP1* polymorphism in the development of metabolic disorders.

To our knowledge, our study was the first to show the contribution of rs1695 and rs614080 of the *GSTP1* gene to the development of metabolic disorders in patients with schizophrenia. Previous genetic studies have focused on mentally healthy individuals. The role of the rs1695 polymorphism in the development of type 2 diabetes mellitus has been widely studied, but data on its contribution remain ambiguous. The results of a meta-analysis (18 studies, 2595 patients with type 2 diabetes mellitus and 2888 healthy individuals) did not reveal associations between the studied polymorphic variant and the risk of type 2 diabetes mellitus, although the authors point out the heterogeneity of the data and the need for further research [48]. *GSTP1* rs1695 is associated with increased risk of obesity and cardiometabolic abnormalities in young adults from Brazil; individuals carrying at least one G allele have a 2.4 times higher chance of being obese compared to those with the A/A genotype [49]. In contrast, a study conducted in a Mexican population found no associations of this SNP with obesity [50]. However, the authors conclude that rs614080 was significantly associated with BMI and *GSTP1* expression levels in adipose tissue [50].

A number of studies have examined associations of polymorphisms of other *GST* classes and metabolic disorders in schizophrenia. The *GSTM1* null genotype, particularly when combined with smoking or the *GSTT1* present genotype, may increase the risk of metabolic abnormalities, such as being overweight and having decreased high-density lipoprotein cholesterol levels [51]. The genotype rs1917760*T/T *GSTK1* was linked to a higher BMI score in male schizophrenia patients, while in female patients this genotype was associated with a lower BMI score [52]. Schizophrenia patients with the *GSTK1* rs1917760*T allele or *GSTM1* deletion had a higher risk of being overweight. This was confirmed for male patients with schizophrenia and/o patients who currently smoke [53].

We found that the type of antipsychotic medication a person takes affected the associations obtained between *GSTP1* rs1695 and BMI. We showed that in patients taking FGAs long-term, carriage of the mutant rs1695*G allele was associated with an increased risk of being overweight. Although SGAs are traditionally associated with an increased incidence of metabolic disorders, FGAs can also lead to weight gain and lipid and glucose imbalances [54,55,56]. One of the possible mechanisms of weight gain against the background of taking FGAs may be oxidative stress, which occurs, among other things, due to decreased GSTP1 activity in individuals with the rs1695*G allele. Interestingly, patients who have taken SGAs for an extended period have shown associations between rs1695 in *GSTP1* and obesity. It has been shown that SGAs do not have a substantial effect on redox processes or demonstrate antioxidant properties [34,44]. It is possible that metabolic disorders caused by SGAs develop in ways that are not related to oxidative stress. SGAs profoundly disrupt glucose and lipid homeostasis in the liver, pancreas, adipose tissue, and skeletal muscle, as well as by acting on hypothalamic centers [43]. Further accumulation of glucose and/or lipids, as well as an increase in the volume of adipose tissue, leads to imbalance in the pro-antioxidant system and mitochondrial dysfunction [57,58]. However, we cannot yet explain this finding with complete certainty, so we are treating it with caution. Further genetic studies in larger patient cohorts are needed to more accurately understand the identified associations.

The present study has some limitations. First, the crucial limitations of our study include the cross-sectional study design and the small sample size. Based on this, the studied groups differed in terms of gender and age composition, duration of the disease, and frequency of smoking. However, these results are typical of a clinical situation where metabolic abnormalities are found in patients with schizophrenia. To reduce the significance of these parameters in the association analysis, we adjusted for gender, age, and smoking. A second limitation is that the study was conducted on a group of chronic patients with schizophrenia who had received long-term antipsychotic treatment; therefore, it is not possible to ascertain the extent to which the patients adhered to the treatment regimen over the prolonged period. Despite the limitations stated above, the results of the present study suggest evidence that *GSTP1* variants can influence metabolic abnormalities in patients with schizophrenia.

## 4. Materials and Methods

### 4.1. Patients

This cross-sectional, case-control study included 639 patients with schizophrenia from the Siberian Federal District (Russia). Patients were recruited from the clinics of the Mental Health Research Institute Tomsk National Research Medical Center, the Tomsk Clinical Psychiatric Hospital, the Hospital of the Siberian State Medical University, the Kemerovo Regional Clinical Psychiatric Hospital, and the N.N. Solodnikova Clinical Psychiatric Hospital of Omsk.

The inclusion criteria in the study were verified diagnosis of schizophrenia according to ICD-10 (International Classification of Diseases 10th revision) criteria, assessed via a structured clinical interview (Structured Clinical Interview for the DSM [SCID]), aged 18 to 55 years, provided informed consent, apparent Caucasian descent, absence of severe organic diseases or somatic disorders in a state of decompensation, and those receiving continuous antipsychotic treatment. The severity of psychopathological symptoms was assessed using the Positive and Negative Syndrome Scale (PANSS). Data were gathered regarding baseline antipsychotic therapy and any concomitant treatments at the time of the examination and over the preceding six months (medicines and doses administered and duration of current medication use). For dose standardization, the daily dose of a chlorpromazine equivalent (CPZeq) was used. MetS was diagnosed according to the International Diabetes Federation (IDF, 2005) criteria [59]. Measurements of the fatty components in the body composition of the participants were conducted using a measuring tape, the non-invasive bioimpedance analysis medical device “Omron BF508”, and an electronic caliper.

Clinical and demographic data of the study sample are presented in Table 11.

### 4.2. Laboratory Methods

After a 12 h fast, blood samples were taken through antecubital venipuncture into vacutainer tubes with EDTA (for DNA) or a clot activator (for serum). DNA from venous peripheral blood was isolated using the standard phenol–chloroform method. Genotyping was performed using real-time polymerase chain reaction (RT-PCR) with the BioMaster UDG HS-qPCR Lo-ROX (2×) PCR kit (BioLabMix, Novosibirsk, Russia) and region-specific primers (DNA-Synthesis, Moscow, Russia) on the amplifier QuantStudio™ 5 Real-Time PCR System (Applied Biosystems, Waltham, MA, USA). The equipment is located at the core facility of Medical Genomics, Tomsk National Research Medical Center, Russian Academy of Sciences.

The polymorphic variants of the *GSTP1* (rs614080 and rs1695) and *GSTO1* (rs49252) genes were selected for genotyping. These gene polymorphisms were chosen for the following reasons:

1. Minor allele frequency of at least 5%.
2. Availability of information on previous studies of this polymorphism.
3. Marker localization.

None of these criteria were decisive, but the presence of at least one item was sufficient for inclusion in the study.

Measurement of the concentration of glucose, total cholesterol, triglycerides, low-density lipoproteins, and high-density lipoproteins in blood serum was performed using standard biochemical methods using commercial kits (Cormay, Łomianki, Poland) on a semi-automatic biochemical analyzer (Cormay Multi Plus, Cormay, Łomianki, Poland).

### 4.3. Statistics

The statistical analysis was conducted using R package version 4.0.4 and SPSS Statistics version 26. A power analysis was conducted with “pwr” package for R. The power of the sample was 0.95 (with a significance level of 0.05 and Cohen’s w = 0.16). The Hardy–Weinberg equilibrium of genotypic frequencies was examined by the chi-squared test. Association analysis was performed using the chi-squared (χ^2^) test. SNPStats online web tool (https://www.snpstats.net/start.htm?q=snpstats/start.htm; accessed on 2 May 2025) was used to examine the inheritance model (data were adjusted for age, smoking, and sex). Comparisons of quantitative data were performed using the Kruskal–Wallis and Mann–Whitney tests. Bonferronni correction was used for multiple comparisons. Differences between the compared groups were considered statistically significant at *p* < 0.05.

## 5. Conclusions

The rs1695 variant of the *GSTP1* is significantly associated with the BMI of schizophrenia patients receiving antipsychotic therapy. In the subgroup of patients receiving FGAs, we found an association of overweight with the rs1695*GG genotype of *GSTP1* (OR: 5.43; 95% CI: 2.24–13.16; *p* < 0.001) in a recessive model of inheritance. Conversely, we demonstrated an association between rs1695*G *GSTP1* and obesity (OR: 0.42; 95% CI: 0.20–0.87; *p* = 0.018) in a dominant inheritance model in patients receiving SGAs. The rs1695*GG variant of *GSTP1* is also associated with higher abdominal fat fold (*p* = 0.037) and visceral fat levels (*p* = 0.047) in patients treated with FGAs. Among patients who received SGAs, we found that carrying the *GSTP1* rs614080*GG variant was associated with higher total cholesterol (*p* = 0.015) and low-density lipoprotein (*p* = 0.036) levels. Our results support the hypothesis that antioxidant status is impaired, particularly with regard to the role of the *GSTP1* gene in antipsychotic-induced metabolic disorders in schizophrenia. These results are preliminary and require further research with larger sample sizes and different ethnic groups.

## Figures and Tables

**Table 1:** Frequencies of alleles and genotypes of polymorphic variants of the *GST* in patients with schizophrenia depending on BMI.

Gene, Polymorphism	Genotype, Allele	Patients with Normal Weight	Patients with Overweight	Patients with Obesity	OR_1_ (95% CI)	χ^2^_1_, *p*-Value_1_	OR_2_ (95% CI)	χ^2^_2_, *p*-Value_2_
*GSTP1* rs614080	A/A	64 (28.7%)	35 (31.2%)	37 (38.5%)	1.13 (0.69–1.85)	5.027; 0.081	1.56 (0.94–2.58)	2.979; 0.225
	G/A	112 (50.2%)	43 (38.4%)	41 (42.7%)	0.62 (0.39–0.98)		0.74 (0.46–1.2)	
	G/G	47 (21.1%)	34 (30.4%)	18 (18.8%)	1.63 (0.97–2.73)		0.86 (0.47–1.58)	
	A	0.536	0.500	0.593	0.87 (0.63–1.2)	0.767; 0.381	1.28 (0.91–1.81)	1.796; 0.18
	G	0.464	0.500	0.407	1.14 (0.83–1.58)		0.78 (0.55–1.1)	
*GSTP1* rs1695	A/A	138 (45%)	64 (43.5%)	65 (53.7%)	0.94 (0.64–1.4)	**6.749; 0.034**	0.8 (0.35–1.82)	2.433; 0.296
	A/G	144 (46.9%)	58 (39.5%)	48 (39.7%)	0.74 (0.49–1.1)		1.28 (0.92–1.79)	
	G/G	25 (8.1%)	25 (17%)	8 (6.6%)	**2.31 (1.28–4.19)**		0.78 (0.56–1.09)	
	A	0.681	0.632	0.732	0.8 (0.59–1.07)	2.167; 0.141	1.42 (0.93–2.17)	2.117; 0.146
	G	0.319	0.368	0.268	1.26 (0.94–1.68)		0.74 (0.49–1.14)	
*GSTO1* rs4925	C/C	160 (52.5%)	68 (46.6%)	62 (51.2%)	0.79 (0.53–1.17)	1.687; 0.43	0.95 (0.62–1.45)	0.64; 0.726
	A/C	122 (40%)	67 (45.9%)	47 (38.8%)	1.27 (0.85–1.89)		0.95 (0.62–1.47)	
	A/A	23 (7.5%)	11 (7.5%)	12 (9.9%)	1 (0.47–2.11)		1.35 (0.65–2.81)	
	C	0.723	0.693	0.711	0.87 (0.64–1.18)	0.875; 0.35	0.92 (0.66–1.27)	0.12; 0.729
	A	0.277	0.307	0.289	1.15 (0.85–1.57)		1.09 (0.79–1.52)	

Notes. Patients with normal weight: BMI < 24.9 kg/cm^2^; patients with overweight: BMI ≥ 25 kg/cm^2^; <30 kg/cm^2^; patients with obesity: BMI ≥ 30 kg/cm^2^; BMI: body mass index; OR: odds ratio; 95% CI: 95% confidence interval; OR_1_, χ^2^_1_, *p*-value_1_ means comparison between groups of patients with normal weight and patients with overweight; OR_2_, χ^2^_2_, *p*-value_2_ means comparison between groups of patients with normal weight and patients with obesity.

**Table 2:** Models of predisposing effect inheritance of rs1695 in the *GSTP1* gene in patients with schizophrenia.

Model	Genotype	Patients with Normal Weight	Patients with Overweight	OR (95% CI)	*p*-Value	AIC	BIC
Codominant	A/A	138 (45%)	64 (43.5%)	1.00	**0.018**	571.5	596.2
	A/G	144 (46.9%)	58 (39.5%)	0.90 (0.58–1.38)			
	G/G	25 (8.1%)	25 (17%)	**2.23 (1.19–4.21)**			
Dominant	A/A	138 (45%)	64 (43.5%)	1.00	0.65	577.3	597.9
	A/G-G/G	169 (55%)	83 (56.5%)	1.10 (0.73–1.63)			
Recessive	A/A-A/G	282 (91.9%)	122 (83%)	1.00	**0.0054**	**569.7**	**590.3**
	G/G	25 (8.1%)	25 (17%)	**2.36 (1.30–4.29)**			
Overdominant	A/A-G/G	163 (53.1%)	89 (60.5%)	1.00	0.17	575.6	596.2
	A/G	144 (46.9%)	58 (39.5%)	0.76 (0.51-1.13)			

Notes. Patients with normal weight: BMI < 24.9 kg/cm^2^; patients with overweight: BMI ≥ 25; <30 kg/cm^2^; OR: odds ratio; 95% CI: 95% confidence interval; AIC: Akaike information criterion; BIC: Bayesian information criterion. The data were adjusted for age, smoking, and sex.

**Table 3:** Association between GST gene polymorphisms and blood lipids and glucose in patients with schizophrenia.

rs614080 *GSTP1*				
**Parameter**	**A/A**	**G/A**	**G/G**	***p*** **-Value**
Fasting glucose (mg/dL)	5 (4.4–5.5)	4.91 (4.3–5.5)	5.1 (4.65–5.6)	0.138
Total cholesterol (mg/dL)	4.4 (3.79–5)	4.5 (3.85–5.11)	4.76 (4.05–5.41)	**0.046 (A/A vs. G/G 0.017)**
Triglyceride (mg/dL)	1.3 (0.9–1.82)	1.26 (0.89–1.8)	1.3 (1–1.75)	0.839
High-density lipoprotein (mg/dL)	1.02 (0.86–1.3)	1 (0.8–1.24)	1.01 (0.8–1.4)	0.493
Low-density lipoprotein (mg/dL)	2.74 (2.19–3.31)	2.64 (2.2–3.33)	3 (2.5–3.8)	**0.028 (A/A vs. G/G 0.031; (A/G vs. G/G 0.011)**
rs4925 *GSTO1*				
**Parameter**	**A/A**	**A/C**	**C/C**	***p*** **-value**
Fasting glucose (mg/dL)	4.9 (4.6–5.4)	5.05 (4.6–5.5)	5 (4.4–5.6)	0.853
Total cholesterol (mg/dL)	4.51 (4.1–5.08)	4.5 (3.8–5.26)	4.51 (3.95–5.28)	0.882
Triglyceride (mg/dL)	1.3 (0.98–2.01)	1.36 (1–1.75)	1.3 (0.94–1.91)	0.955
High-density lipoprotein (mg/dL)	1.02 (0.79–1.27)	1 (0.82–1.3)	1.08 (0.8–1.3)	0.568
Low-density lipoprotein (mg/dL)	2.75 (2.41–3.28)	2.71 (2.2–3.59)	2.9 (2.29–3.5)	0.861
rs1695 *GSTP1*				
**Parameter**	**A/A**	**A/G**	**G/G**	***p*** **-value**
Fasting glucose (mg/dL)	5.1 (4.5–5.5)	5 (4.5–5.52)	4.9 (4.34–5.35)	0.367
Total cholesterol (mg/dL)	4.48 (3.89–5.22)	4.5 (3.81–5.27)	4.7 (4–5.25)	0.923
Triglyceride (mg/dL)	1.3 (0.89–1.81)	1.31 (1–1.95)	1.31 (0.95–1.7)	0.536
High-density lipoprotein (mg/dL)	1.05 (0.82–1.32)	1.02 (0.82–1.29)	0.96 (0.8–1.3)	0.634
Low-density lipoprotein (mg/dL)	2.77 (2.2–3.61)	2.79 (2.27–3.41)	2.98 (2.39–3.7)	0.562

Notes. Comparisons of data between the three groups were performed using the Kruskal–Wallis test. Pairwise comparisons were performed using the Mann–Whitney test with Bonferroni correction.

**Table 4:** Association between *GST* gene polymorphisms and anthropometric parameters in patients with schizophrenia.

rs614080 *GSTP1*				
**Parameter**	**A/A**	**G/A**	**G/G**	***p*** **-Value**
Waist circumference, cm	88 (79.5–98)	85 (78–97)	87 (80–97.5)	0.443
The body fat percentage result	30.05 (20.8–40.1)	27.6 (20.45–37.75)	29.6 (19.3–39.9)	0.665
Visceral fat level	6 (5–9)	7 (4–9)	7 (5–10)	0.455
Total fat fold	76 (52–103)	74 (55.5–101)	78 (50–106)	0.994
Abdominal fat fold	29 (20–38)	32 (21–40)	29 (23–37)	0.664
rs4925 *GSTO1*				
**Parameter**	**A/A**	**A/C**	**C/C**	***p*** **-value**
Waist circumference, cm	86 (81–98)	85 (78.5–96)	85 (77–97)	0.542
The body fat percentage result	26.15 (19.8–41.6)	28.9 (21.35–37.6)	28.9 (18.7–38.3)	0.968
Visceral fat level	6.5 (4–8)	7 (4.5–9)	6.5 (4–9)	0.761
Total fat fold	65 (49–99)	70.5 (53–101)	78 (51–103)	0.668
Abdominal fat fold	30 (22–44)	28.5 (19.5–39)	30.5 (21–38)	0.724
rs1695 *GSTP1*				
**Parameter**	**A/A**	**A/G**	**G/G**	***p*** **-value**
Waist circumference, cm	85 (78–97)	85 (78–96)	86 (78–95.5)	0.973
The body fat percentage result	29.1 (20.75–38.3)	27.4 (18.7–36.4)	30.5 (25.2–37.95)	0.275
Visceral fat level	6 (4–8)	6 (4–9)	8 (6–10)	0.16
Total fat fold	74.5 (48–99)	72 (53–101)	85 (71–103)	0.358
Abdominal fat fold	29 (21–38)	28 (18–37)	35 (27.5–43)	**0.023** **(A/A vs. G/G 0.027; A/G vs. G/G 0.007)**

Notes. Comparisons of data between the three groups were performed using the Kruskal–Wallis test. Pairwise comparisons were performed using the Mann–Whitney test with Bonferroni correction.

**Table 5:** The frequencies of alleles and genotypes of polymorphic variants of the *GST* in patients with schizophrenia receiving FGAs depending on BMI.

Gene, Polymorphism	Genotype, Allele	Patients with Normal Weight	Patients with Overweight	Patients with Obesity	OR_1_ (95% CI)	χ^2^_1_, *p*-Value_1_	OR_2_ (95% CI)	χ^2^_2_, *p*-Value_2_
*GSTP1* rs614080	A/A	41 (28.9%)	14 (25%)	20 (35.1%)	0.82 (0.41–1.66)	4.401; 0.111	1.33 (0.69–2.56)	0.728; 0.695
	G/A	71 (50%)	22 (39.3%)	26 (45.6%)	0.65 (0.34–1.21)		0.84 (0.45–1.55)	
	G/G	30 (21.1%)	20 (35.7%)	11 (19.3%)	2.07 (1.05–4.09)		0.89 (0.41–1.93)	
	A	0.539	0.446	0.579	0.69 (0.44–1.07)	2.739; 0.098	1.18 (0.76–1.83)	0.532; 0.466
	G	0.461	0.554	0.421	1.45 (0.93–2.25)		0.85 (0.55–1.32)	
*GSTP1* rs1695	A/A	85 (45.2%)	31 (40.8%)	36 (48.6%)	0.83 (0.49–1.43)	**13.792; 0.001**	1.15 (0.67–1.97)	0.586; 0.746
	A/G	93 (49.5%)	29 (38.2%)	33 (44.6%)	0.63 (0.37–1.09)		0.82 (0.48–1.41)	
	G/G	10 (5.3%)	16 (21.1%)	5 (6.8%)	**4.75 (2.04–11.02)**		1.29 (0.43–3.91)	
	A	0.699	0.599	0.709	**0.64 (0.43–0.95)**	**4.976; 0.026**	1.05 (0.69–1.59)	0.051; 0.822
	G	0.301	0.401	0.291	**1.56 (1.05–2.31)**		0.95 (0.63–1.45)	
*GSTO1* rs4925	C/C	93 (49.5%)	36 (47.4%)	40 (54.8%)	0.92 (0.54–1.57)	0.097; 0.953	1.24 (0.72–2.13)	0.74; 0.691
	A/C	81 (43.1%)	34 (44.7%)	29 (39.7%)	1.07 (0.63–1.83)		0.87 (0.5–1.51)	
	A/A	14 (7.4%)	6 (7.9%)	4 (5.5%)	1.07 (0.39–2.88)		0.72 (0.23–2.27)	
	C	0.710	0.697	0.747	0.94 (0.62–1.42)	0.085; 0.771	1.2 (0.78–1.86)	0.694; 0.405
	A	0.290	0.303	0.253	1.06 (0.7–1.6)		0.83 (0.54–1.28)	

Notes. Patients with normal weight: BMI < 24.9 kg/cm^2^; patients with overweight: BMI ≥ 25 kg/cm^2^; <30 kg/cm^2^; patients with obesity: BMI ≥ 30 kg/cm^2^; FGA: first-generation antipsychotics; BMI: body mass index; OR: odds ratio; 95% CI: 95% confidence interval; OR_1_, χ^2^_1_, *p*-value_1_ means comparison between groups of patients with normal weight and patients with overweight; OR_2_, χ^2^_2_, *p*-value_2_ means comparison between groups of patients with normal weight and patients with obesity.

**Table 6:** Models of predisposing effect inheritance of rs1695 in the *GSTP1* gene in patients with schizophrenia receiving FGAs.

Model	Genotype	Patients with Normal Weight	Patients with Overweight	OR (95% CI)	*p*-Value	AIC	BIC
Codominant	A/A	85 (45.7%)	31 (41.9%)	1.00	**6.00 × 10^−4^**	**299.6**	**321**
	A/G	92 (49.5%)	27 (36.5%)	0.87 (0.47–1.59)			
	G/G	9 (4.8%)	16 (21.6%)	**5.07 (2.00–12.87)**			
Dominant	A/A	85 (45.7%)	31 (41.9%)	1.00	0.42	311.9	329.7
	A/G-G/G	101 (54.3%)	43 (58.1%)	1.26 (0.72–2.20)			
Recessive	A/A-A/G	177 (95.2%)	58 (78.4%)	1.00	**1.00 × 10^−4^**	**297.8**	**315.6**
	G/G	9 (4.8%)	16 (21.6%)	**5.43 (2.24–13.16)**			
Overdominant	A/A-G/G	94 (50.5%)	47 (63.5%)	1.00	0.1	309.9	327.7
	A/G	92 (49.5%)	27 (36.5%)	0.63 (0.36–1.10)			

Notes. Patients with normal weight: BMI < 24.9 kg/cm^2^; patients with overweight: BMI ≥ 25 kg/cm^2^; <30 kg/cm^2^; OR: odds ratio; 95% CI: 95% confidence interval; AIC: Akaike information criterion; BIC: Bayesian information criterion. The data were adjusted for age, smoking, and sex.

**Table 7:** Association between GST gene polymorphisms and anthropometric parameters in patients with schizophrenia receiving FGAs.

rs614080 *GSTP1*				
**Parameter**	**A/A**	**G/A**	**G/G**	***p*** **-Value**
Waist circumference, cm	84 (79–95)	85 (78–95)	86 (80–99)	0.537
The body fat percentage result	25.8 (18–38.1)	27.4 (19.5–37.5)	26.8 (18–37.6)	0.838
Visceral fat level	6 (4–9)	7 (4–9)	7 (5–10)	0.468
Total fat fold	65 (48–92)	72.5 (49–101)	67 (47.5–90)	0.662
Abdominal fat fold	27 (19.5–37.5)	26 (19–36.5)	26 (19–31.5)	0.755
rs4925 *GSTO1*				
**Parameter**	**A/A**	**A/C**	**C/C**	***p*** **-value**
Waist circumference, cm	84 (78.5–92.5)	84 (79–95)	85 (78–97)	0.962
The body fat percentage result	25.9 (20.7–37.8)	26.3 (19.1–37.6)	27.3 (17.6–36)	0.956
Visceral fat level	6 (4–7)	6 (5–8)	7 (4–9)	0.682
Total fat fold	60 (47–88)	67 (48–99)	73 (47.5–97)	0.735
Abdominal fat fold	27 (22–34)	26 (19–37)	26.5 (18–35)	0.846
rs1695 *GSTP1*				
**Parameter**	**A/A**	**A/G**	**G/G**	***p*** **-value**
Waist circumference, cm	83 (78–95)	85 (78–98)	89 (82–95)	0.328
The body fat percentage result	26.2 (18.65–35.8)	25.8 (18.2–37.5)	30.55 (25.45–38.4)	0.200
Visceral fat level	6 (4–8)	6 (4–8)	8 (6–10)	**0.047 (A/A vs. G/G 0.017; A/G vs. G/G 0.016)**
Total fat fold	67 (46–91.5)	68 (50–100)	85 (53–102)	0.350
Abdominal fat fold	26.5 (20–35.5)	25 (16–34)	33 (27–41)	**0.037 (A/A vs. G/G 0.031; (A/G vs. G/G 0.038)**

Notes. Comparisons of data between the three groups were performed using the Kruskal–Wallis test. Pairwise comparisons were performed using the Mann–Whitney test with Bonferroni correction.

**Table 8:** The frequencies of alleles and genotypes of polymorphic variants of the GST gene in patients with schizophrenia receiving SGAs depending on BMI.

Gene, Polymorphism	Genotype, Allele	Patients with Normal Weight	Patients with Overweight	Patients with Obesity	OR_1_ (95% CI)	χ ^2^_1_, *p*-Value_1_	OR_2_ (95% CI)	χ^2^_2_, *p*-Value_2_
*GSTP1* rs614080	A/A	23 (27.7%)	21 (36.8%)	17 (42.5%)	1.52 (0.74–3.13)	2.091; 0.351	1.93 (0.88–4.25)	2.812; 0.245
	G/A	42 (50.6%)	22 (38.6%)	15 (37.5%)	0.61 (0.31–1.22)		0.59 (0.27–1.27)	
	G/G	18 (21.7%)	14 (24.6%)	8 (20%)	1.18 (0.53–2.61)		0.9 (0.35–2.3)	
	A	0.530	0.561	0.613	1.13 (0.7–1.83)	0.267; 0.606	1.4 (0.81–2.41)	1.485; 0.223
	G	0.470	0.439	0.388	0.88 (0.55–1.42)		0.71 (0.41–1.23)	
*GSTP1* rs1695	A/A	42 (40.8%)	29 (43.9%)	29 (61.7%)	1.14 (0.61–2.13)	0.189; 0.91	2.34 (1.15–4.75)	6.026; 0.054
	A/G	47 (45.6%)	28 (42.4%)	15 (31.9%)	0.88 (0.47–1.64)		0.56 (0.27–1.15)	
	G/G	14 (13.6%)	9 (13.6%)	3 (6.4%)	1 (0.41–2.47)		0.43 (0.12–1.59)	
	A	0.636	0.652	0.777	1.07 (0.68–1.69)	0.085; 0.77	**1.99 (1.13–3.49)**	**5.87; 0.015**
	G	0.364	0.348	0.223	0.93 (0.59–1.48)		**0.5 (0.29–0.88)**	
*GSTO1* rs4925	C/C	56 (54.4%)	28 (42.4%)	22 (45.8%)	0.62 (0.33–1.15)	2.355; 0.308	0.71 (0.36–1.41)	3.452; 0.178
	A/C	40 (38.8%)	33 (50%)	18 (37.5%)	1.58 (0.84–2.94)		0.95 (0.47–1.91)	
	A/A	7 (6.8%)	5 (7.6%)	8 (16.7%)	1.12 (0.34–3.7)		2.74 (0.93–8.07)	
	C	0.738	0.674	0.646	0.74 (0.46–1.19)	1.591; 0.207	0.65 (0.38–1.09)	2.686; 0.101
	A	0.262	0.326	0.354	1.36 (0.84–2.19)		1.54 (0.92–2.6)	

Notes. Patients with normal weight: BMI < 24.9 kg/cm^2^; patients with overweight: BMI ≥ 25; <30 kg/cm^2^; patients with obesity: BMI ≥ 30 kg/cm^2^; SGA: second-generation antipsychotics; BMI: body mass index; OR: odds ratio; 95% CI: 95% confidence interval; OR_1_, χ^2^_1_, *p*-value_1_ means comparison between groups of patients with normal weight and patients with overweight; OR_2_, χ^2^_2_, *p*-value_2_ means comparison between groups of patients with normal weight and patients with obesity.

**Table 9:** Models of predisposing effect inheritance of rs1695 in the *GSTP1* gene in patients with schizophrenia receiving SGAs.

Model	Genotype	Patients with Normal Weight	Patients with Obesity	OR (95% CI)	*p*-Value	AIC	BIC
Codominant	A/A	42 (40.8%)	29 (61.7%)	1.00	0.048	181.9	200
	A/G	47 (45.6%)	15 (31.9%)	0.46 (0.21–1.01)			
	G/G	14 (13.6%)	3 (6.4%)	0.28 (0.07–1.10)			
Dominant	A/A	42 (40.8%)	29 (61.7%)	1.00	**0.018**	**180.4**	**195.5**
	A/G-G/G	61 (59.2%)	18 (38.3%)	**0.42 (0.20–0.87)**			
Recessive	A/A-A/G	89 (86.4%)	44 (93.6%)	1.00	0.14	183.8	198.8
	G/G	14 (13.6%)	3 (6.4%)	0.39 (0.10–1.48)			
Overdominant	A/A-G/G	56 (54.4%)	32 (68.1%)	1.00	0.14	183.8	198.8
	A/G	47 (45.6%)	15 (31.9%)	0.57 (0.27–1.22)			

Notes. Patients with normal weight: BMI < 24.9 kg/cm^2^; patients with obesity: BMI ≥ 30 kg/cm^2^; OR: odds ratio; 95% CI: 95% confidence interval; AIC: Akaike information criterion; BIC: Bayesian information criterion. The data were adjusted for age, smoking, and sex.

**Table 10:** Association between *GST* gene polymorphisms and blood lipids and glucose in patients with schizophrenia receiving SGAs.

rs614080 *GSTP1*				
**Parameter**	**A/A**	**G/A**	**G/G**	***p*** **-Value**
Fasting glucose (mg/dL)	5 (4.49–5.4)	4.9 (4.3–5.4)	5.19 (4.7–5.6)	0.208
Total cholesterol (mg/dL)	4.45 (3.83–5.09)	4.31 (3.78–4.86)	5.05 (4.14–5.72)	**0.015 (A/A vs. G/G 0.021; A/G vs. G/G 0.004)**
Triglyceride (mg/dL)	1.4 (1.1–1.9)	1.27 (0.86–2.01)	1.37 (1.2–1.8)	0.620
High-density lipoprotein (mg/dL)	1 (0.8–1.29)	0.98 (0.8–1.2)	1.09 (0.9–1.45)	0.100
Low-density lipoprotein (mg/dL)	2.67 (1.99–3.31)	2.52 (2.17–2.99)	3.01 (2.31–3.99)	**0.036 (A/A vs. G/G 0.030; A/G vs. G/G 0.015)**
rs4925 *GSTO1*				
**Parameter**	**A/A**	**A/C**	**C/C**	***p*** **-value**
Fasting glucose (mg/dL)	4.86 (4.5–5.25)	5 (4.51–5.44)	4.98 (4.5–5.5)	0.722
Total cholesterol (mg/dL)	4.63 (4.1–5.08)	4.43 (3.77–5.32)	4.6 (4–5.33)	0.471
Triglyceride (mg/dL)	1.63 (1.02–2.07)	1.4 (1.03–1.9)	1.3 (0.9–1.94)	0.466
High-density lipoprotein (mg/dl)	0.9 (0.68–1.1)	1.01 (0.84–1.2)	1.1 (0.87–1.36)	0.119
Low-density lipoprotein (mg/dL)	2.84 (2.38–3.28)	2.6 (2.12–3.16)	2.74 (2.29–3.36)	0.571
rs1695 *GSTP1*				
**Parameter**	**A/A**	**A/G**	**G/G**	***p*** **-value**
Fasting glucose (mg/dL)	5.1 (4.6–5.5)	4.9 (4.5–5.4)	4.93 (4.12–5.27)	0.212
Total cholesterol (mg/dL)	4.58 (4.02–5.42)	4.49 (3.8–5.11)	4.5 (3.72–5.28)	0.482
Triglyceride (mg/dL)	1.5 (0.9–1.91)	1.3 (1.01–2)	1.4 (0.86–1.95)	0.891
High-density lipoprotein (mg/dL)	1.1 (0.82–1.32)	1.07 (0.86–1.3)	0.9 (0.8–1.2)	0.270
Low-density lipoprotein (mg/dL)	2.7 (2.2–3.4)	2.67 (2.1–3.11)	2.77 (2.3–3.3)	0.573

Notes. Comparisons of data between the three groups were performed using the Kruskal–Wallis test. Pairwise comparisons were performed using the Mann–Whitney test with Bonferroni correction.

**Table 11:** Clinical and demographic parameters of the patients.

Sample size, n	639
Sex, n (%)	Male: 321 (50.2%)
	Female: 318 (49.8%)
Age, years, Me (Q1; Q3)	39 (32; 50)
Age of manifestation, years, Me (Q1; Q3)	24 (20; 30)
Duration of disease, years, Me (Q1; Q3)	13 (7; 21)
PANSS, total score, Me (Q1; Q3)	101 (91; 110)
Chlorpromazine equivalent doses, Me (Q1; Q3)	429 (225; 750)
Antipsychotic generation for basic therapy, n (%)	First generation: 392 (61.4)
	Second generation: 247 (38.6)
Chlorpromazine	33 (5.5%)
Haloperidol	216 (35.8%)
Trifluoperazine	32 (5.3%)
Zuclopenthixol	20 (3.3%)
Risperidone	95 (15.7%)
Clozapine	34 (5.6%)
Olanzapine	32 (5.3%)
Quetiapine	42 (7.0%)
Smoking, n (%)	Yes: 371 (58.1%)
	No: 268 (41.9%)
Metabolic syndrome, n (%)	Yes: 164 (25.7%)
	No: 475 (74.3%)
Body mass index, kg/cm^2^	Normal (≤24.9): 312 (52.8%)
	Overweight (25–29.9): 153 (25.9%)
	Obesity (≥30): 126 (21.3%)

## Data Availability

The datasets generated for this study will not be made publicly available, but they are available upon reasonable request from Svetlana A. Ivanova (ivanovaniipz@gmail.com) following approval of the Board of Directors of the MHRI, in line with local guidelines and regulations.

## Supplementary Materials

The following supporting information can be downloaded at https://www.mdpi.com/article/10.3390/ph18070941/s1, Table S1. SNPs’ characteristics and results of the Hardy–Weinberg equilibrium test; Table S2. The frequencies of alleles and genotypes of *GST* polymorphic variants gene in the patients with and without MetS; Table S3. The frequencies of alleles and genotypes of *GST* polymorphic variants in the patients who received FGAs; Table S4. Association between *GST* gene polymorphisms and blood lipids and glucose in patients who received FGAs; Table S5. The frequencies of alleles and genotypes of *GST* polymorphic variants in the patients who received SGAs; Table S6. Association between *GST* gene polymorphisms and anthropometric parameters in patients who received SGAs. Table S7. Association between *GST* gene polymorphisms and anthropometric parameters in the groups of patients received second-generation antipsychotics.

## Abbreviations

The following abbreviations are used in this manuscript:

BMI	Body mass index
CI	Confidence interval
FGA	First-generation antipsychotic
GST	Glutathione-S-transferase
MetS	Metabolic syndrome
OR	Odds ratio
SGA	Second-generation antipsychotic
SNP	Single-nucleotide polymorphism
