# Supplementary material for: The Role of GST Gene Polymorphic Variants in Antipsychotic-Induced Metabolic Disorders in Schizophrenia: A Pilot Study
Source: Pharmaceuticals (Basel). 2025 Jun 21;18(7):941. doi: 10.3390/ph18070941 (PMC12300524; doi:10.3390/ph18070941)
Supplement: Supplementary file 1 [file pharmaceuticals-18-00941-s001.zip › pharmaceuticals-3672830-supplementary.pdf]

Table S1. SNPs' characteristics and results of the Hardy–Weinberg equilibrium test.

**Gene**	***GSTP1***	***GSTP1***	***GSTO1***
SNP	rs614080	rs1695	rs4925
Chromosome	chr11	chr11	chr10
Position	67579816	67585218	104263031
Alleles	G/A	A/G	C/A
Type	regulatory region variant	missense variant	missense variant
MAF (study)	0.48	0.32	0.29
MAF (Europe)	0.494	0.331	0.337
MAF (African)	0.065	0.480	0.069
MAF (East Asian)	0.676	0.179	0.130
MAF (South Asian)	0.636	0.294	0.186
MAF (American)	0.331	0.476	0.207
MAF (Global)	0.423	0.353	0.177
p HWE	0.131	0.904	0.798

Notes. p HWE: the p value of the Hardy–Weinberg equilibrium test; MAF (study): our found MAF values; MAF (Ensembl; EUR): MAF from the Ensemble database on the basis of the "1000 Genomes" project.

Table S2. The frequencies of alleles and genotypes of polymorphic variants GST gene in the compared groups

Gene, polymorphism	Genotype. allele	Patients with MetS	Patients without MetS	OR [95 CI]	X^2, p-value
GSTP1 rs614080	A/A	40 (30.8%)	108 (31%)	0.99 [0.64 - 1.53]	0.735, 0.692
	G/A	57 (43.8%)	164 (47.1%)	0.88 [0.58 - 1.31]	
	G/G	33 (25.4%)	76 (21.8%)	1.22 [0.76 - 1.95]	
	A	0.52	0.54	0.93 [0.7 - 1.23]	0.277, 0.599
	G	0.47	0.45	1.08 [0.81 - 1.44]	
GSTP1 rs1695	A/A	79 (49.1%)	214 (45.5%)	1.15 [0.81 - 1.65]	0.664, 0.717
	A/G	67 (41.6%)	206 (43.8%)	0.91 [0.64 - 1.31]	
	G/G	15 (9.3%)	50 (10.6%)	0.86 [0.47 - 1.58]	
	A	0.699	0.674	1.12 [0.85 - 1.47]	0.651, 0.42
	G	0.301	0.326	0.89 [0.68 - 1.18]	
GSTO1 rs4925	C/C	89 (55.3%)	230 (49.3%)	1.27 [0.89 - 1.83]	1.982, 0.371
	A/C	59 (36.6%)	200 (42.8%)	0.77 [0.53 - 1.12]	
	A/A	13 (8.1%)	37 (7.9%)	1.02 [0.53 - 1.97]	
	C	0.736	0.707	1.16 [0.87 - 1.54]	1.013, 0.314
	A	0.264	0.293	0.86 [0.65 - 1.15]	

Notes. OR: odds ratio; 95% CI: 95% confidence interval.

Table S3. Interaction analysis with covariates

	Codominant	Dominant	Recessive	Overdominant
**overal group**				
gender	p=0.37	p=0.4	p=0.19	p=0.89
smoking	p=0.75	p=0.56	p=0.48	p=0.99
**patients received first-generation antipsychotics**				
gender	p=0.48	p=0.17	p=0.63	p=0.37
smoking	p=0.41	p=0.27	p=0.23	p=0.91
**patients received second-generation antipsychotics**				
gender	p=0.72	p=0.41	p=0.63	p=0.72
smoking	p=0.48	p=0.22	p=0.75	p=0.30

Table S4. The frequencies of alleles and genotypes of polymorphic variants GST gene in the groups of patients received first-generation antipsychotics

Gene, polymorphism	Genotype. allele	Patients with MetS	Patients without MetS	OR [95 CI]	X^2, p-value
GSTP1 rs614080	A/A	20 (30,3%)	63 (29%)	1.06 [0.58 - 1.94]	0.124, 0.949
	G/A	30 (45,5%)	104 (47,9%)	0.91 [0.52 - 1.57]	
	G/G	16 (24,2%)	50 (23%)	1.07 [0.56 - 2.04]	
	A	0.530	0.530	1 [0.68 - 1.48]	0.001, 0.994
	G	0.470	0.470	1 [0.68 - 1.48]	
GSTP1 rs1695	A/A	42 (47.7%)	129 (46.1%)	1.07 [0.66 - 1.73]	0.074, 0963
	A/G	38 (43.2%)	125 (44.6%)	0.94 [0.58 - 1.53]	
	G/G	8 (9.1%)	26 (9.3%)	0.98 [0.43 - 2.24]	
	A	0,693	0,684	1.04 [0.72 - 1.51]	0.053, 0.818
	G	0,307	0,316	0.96 [0.66 - 1.38]	
GSTO1 rs4925	C/C	51 (58%)	134 (48%)	1.49 [0.92 - 2.42]	2.711, 0.258
	A/C	32 (36,4%)	123 (44,1%)	0.72 [0.44 - 1.19]	
	A/A	5 (5,7%)	22 (7,9%)	0.7 [0.26 - 1.92]	
	C	0,761	0,701	1.36 [0.92 - 2.01]	2.416, 0.120
	A	0,239	0,299	0.73 [0.5 - 1.09]	

Notes. OR: odds ratio; 95% CI: 95% confidence interval.

Table S5. Association between GST gene polymorphisms and blood lipids and glucose in patients received first-generation antipsychotics

rs614080 *GSTP1*				
**Parameter**	**A/A**	**G/A**	**G/G**	**p-value**
Fasting glucose (mg/dl)	5 (4.3 - 5.7)	4.97 (4.3 - 5.5)	5.08 (4.6 - 5.6)	0.416
Total cholesterol(mg/dl)	4.39 (3.78 - 4.84)	4.52 (3.98 - 5.25)	4.6 (3.91 - 5.1)	0.264
Triglyceride (mg/dl)	1.22 (0.82 - 1.81)	1.25 (0.91 - 1.71)	1.23 (0.95 - 1.75)	0.902
High-density lipoprotein (mg/dl)	1.02 (0.86 - 1.32)	1.02 (0.79 - 1.27)	0.92 (0.78 - 1.26)	0.248
Low-density lipoprotein (mg/dl)	2.88 (2.36 - 3.49)	2.85 (2.2 - 3.57)	3 (2.5 - 3.57)	0.597
**rs4925 *GSTO1***				
**Parameter**	**A/A**	**A/C**	**C/C**	**p-value**
Fasting glucose (mg/dl)	4.91 (4.6 - 5.5)	5.1 (4.6 - 5.6)	5 (4.31 - 5.6)	0.684
Total cholesterol (mg/dl)	4.46 (4 - 4.9)	4.5 (3.81 - 5.2)	4.49 (3.88 - 5.18)	0.923
Triglyceride (mg/dl)	1.14 (0.96 - 1.5)	1.35 (0.9 - 1.7)	1.29 (0.95 - 1.9)	0.696
High-density lipoprotein (mg/dl)	1.06 (0.87 - 1.28)	0.99 (0.82 - 1.3)	1.02 (0.79 - 1.3)	0.926
Low-density lipoprotein (mg/dl)	2.68 (2.42 - 2.96)	2.87 (2.34 - 3.7)	2.94 (2.27 - 3.51)	0.479
**rs1695 *GSTP1***				
**Parameter**	**A/A**	**A/G**	**G/G**	**p-value**
Fasting glucose (mg/dl)	5.08 (4.46 - 5.5)	5.1 (4.45 - 5.6)	4.85 (4.44 - 5.5)	0.542
Total cholesterol (mg/dl)	4.4 (3.82 - 5.14)	4.51 (3.86 - 5.27)	4.8 (4.12 - 5.2)	0.573
Triglyceride (mg/dl)	1.23 (0.83 - 1.73)	1.36 (1 - 1.83)	1.26 (0.98 - 1.55)	0.266
High-density lipoprotein (mg/dl)	1.02 (0.82 - 1.31)	1 (0.8 - 1.28)	1.1 (0.76 - 1.41)	0.835
Low-density lipoprotein (mg/dl)	2.84 (2.26 - 3.69)	2.9 (2.3 - 3.49)	3.14 (2.51 - 3.72)	0.708

Notes. Comparisons of data between the three groups were performed using the Kruskal-Wallis test.

Table S6. The frequencies of alleles and genotypes of polymorphic variants GST gene in the groups of patients received second-generation antipsychotics

Gene, polymorphism	Genotype. allele	Patients with MetS	Patients without MetS	OR [95 CI]	X^2, p-value
GSTP1 rs614080	A/A	20 (31.2%)	45 (34.6%)	1.16 [0.61 - 2.21]	1.329, 0.515
	G/A	27 (42.2%)	60 (46.2%)	1.17 [0.64 - 2.15]	
	G/G	17 (26.6%)	25 (19.2%)	0.66 [0.33 - 1.33]	
	A	0,523	0,577	1.24 [0.81 - 1.9]	0.995, 0.318
	G	0,477	0,423	0.81 [0.53 - 1.23]	
GSTP1 rs1695	A/A	36 (52.2%)	68 (42%)	1.51 [0.86 - 2.66]	2.095, 0.351
	A/G	26 (37.7%)	72 (44.4%)	0.76 [0.42 - 1.35]	
	G/G	7 (10.1%)	22 (13.6%)	0.72 [0.29 - 1.77]	
	A	0,710	0,642	1.37 [0.89 - 2.11]	2.011, 0.156
	G	0,290	0,358	0.73 [0.48 - 1.13]	
GSTO1 rs4925	C/C	36 (52.2%)	78 (47.9%)	1.19 [0.68 - 2.09]	0.718, 0.699
	A/C	26 (37.7%)	71 (43.6%)	0.78 [0.44 - 1.4]	
	A/A	7 (10.1%)	14 (8.6%)	1.2 [0.46 - 3.12]	
	C	0,710	0,696	1.07 [0.69 - 1.65]	0.088, 0.766
	A	0,290	0,304	0.94 [0.6 - 1.45]	

Notes. OR: odds ratio; 95% CI: 95% confidence interval.

Table S7. Association between GST gene polymorphisms and anthropometric parameters in the groups of patients received second-generation antipsychotics

		**rs614080 *GSTP1***		
**Parameter**	**A/A**	**G/A**	**G/G**	**p-value**
Waist circumference, cm	91 (80 - 102)	86 (78 - 98)	87 (79 - 97)	0.493
The body fat percentage result	33.4 (24.2 - 40.1)	28.5 (21.6 - 38.5)	32.6 (27.3 - 42.25)	0.151
Visceral fat level	6.5 (5 - 9)	6.5 (4 - 9)	6.5 (4 - 9)	0.797
Total fat fold	84 (62 - 107)	75 (59 - 101)	92.5 (66 - 113)	0.432
Abdominal fat fold	29.5 (21 - 36)	37.5 (25.5 - 42.5)	37 (28 - 41)	0.105
		**rs4925 *GSTO1***		
**Parameter**	**A/A**	**A/C**	**C/C**	**p-value**
Waist circumference, cm	88 (84 - 107)	88 (78 - 97)	87 (76 - 99)	0.278
The body fat percentage result	26.4 (19.35 - 45.05)	30 (23.55 - 37.45)	31.15 (23.8 - 39.5)	0.978
Visceral fat level	8 (4 - 8)	7 (4 - 9)	6 (4.5 - 9)	0.962
Total fat fold	75.5 (57.5 - 122.5)	75.5 (60.5 - 101.5)	87 (60 - 110)	0.505
Abdominal fat fold	30 (21 - 44)	31 (21 - 40)	36 (29 - 42)	0.267
		**rs1695 *GSTP1***		
**Parameter**	**A/A**	**A/G**	**G/G**	**p-value**
Waist circumference, cm	89.5 (80 - 101.5)	84 (76 - 96)	85 (75 - 97.5)	0.074
The body fat percentage result	33.25 (23.55 - 41.7)	28.6 (22.4 - 35.65)	30.25 (25.2 - 34.5)	0.227
Visceral fat level	7 (5 - 8)	6 (4 - 9.5)	7 (4 - 9)	0.727
Total fat fold	83.5 (62 - 115)	77 (56 - 104)	84.5 (74 - 113)	0.604
Abdominal fat fold	35.25 (24 - 40)	31 (22 - 40)	38.5 (28 - 44)	0.420

Notes. Comparisons of data between the three groups were performed using the Kruskal-Wallis test.
